# The Global Polio Eradication Initiative Stop Transmission of Polio (STOP) Program — 1999–2013

**Published:** 2013-06-21

**Authors:** Polio Eradication Dept, World Health Organization, Global Immunization Program, UNICEF, Bill and Melinda Gates Foundation, Global Immunization Div, Center for Global Health, CDC

In 1988, the Global Polio Eradication Initiative (GPEI) was established through a partnership between the World Health Organization (WHO), Rotary International, CDC, and the United Nations Children’s Fund (UNICEF). By 2012, the annual incidence of polio had decreased by >99%, compared with 1988, and the number of countries in which wild poliovirus (WPV) circulation has never been interrupted was reduced to three: Afghanistan, Nigeria, and Pakistan (1). However, because of the persistence of endemic WPV transmission and recurring outbreaks in polio-free countries after the original polio eradication target date of 2000 (2–4), the World Health Assembly in 2012 declared the completion of polio eradication a programmatic emergency (5). A key component of GPEI is the Stop Transmission of Polio (STOP) program, which was developed and initiated by CDC with WHO in 1999 to mobilize additional human resources and technical assistance for countries affected by WPV transmission. During 1999–2013, 1,563 volunteers were identified, trained, and deployed for 2,221 assignments in 69 countries. The number of volunteers increased from 90–120 per year during 1999–2011 to 287 in 2012 and 378 in 2013, and the number of volunteer person-months in the field per year increased from 273 in 1999 to 1,456 in 2012. The STOP program has aided GPEI by strengthening the capacity of country-level immunization programs and by allowing a large cohort of volunteers to gain valuable field experience that prepares them well for subsequent work as staff members of WHO, UNICEF, and other public health agencies.

## Development and Implementation of the STOP Program

A key factor contributing to the success of the global smallpox eradication program in the 1970s was the deployment of international public health field staff to assist national programs with smallpox outbreak investigation, surveillance, and planning of vaccination activities in endemic countries (6). In 1999, STOP was developed to support GPEI in a similar fashion. STOP teams typically comprise a diverse mix of health professionals, including nurses, physicians, epidemiologists, veterinarians, and information systems and communication specialists. The first STOP volunteers were recruited from CDC staff; however, recruitment was rapidly expanded to include public health professionals from around the world to meet the demand for assistance. STOP volunteers receive daily subsistence allowances, but no other financial remuneration. (Recently, only a small proportion of volunteers are otherwise supported by their employers.) WHO and UNICEF are responsible for assigning volunteers to specific countries to provide technical assistance and training for immunization programs at national, state/province, or district levels and volunteers are supervised by WHO and UNICEF country teams during the assignment.

The initial objectives of STOP field assignments were to conduct and support acute flaccid paralysis (AFP) surveillance and to plan, monitor, and evaluate large-scale supplementary polio immunization campaigns. Field assignment objectives were expanded in 2002 to support accelerated progress toward measles mortality reduction and development of data management systems for disease surveillance. The objectives were further expanded in 2003 to support strengthening routine childhood immunization activities, a key GPEI strategic component; in 2006 to support polio program communications and social mobilization at UNICEF country offices; and in 2011 to support the management needs of immunization and eradication teams at country level.

The STOP program recruits and deploys three types of volunteers: field staff and data managers who work with WHO country teams, and communications officers who work with UNICEF teams. Since 2009, an “enhanced” STOP program component has placed senior, experienced volunteers at the district level in the highest priority areas. All volunteers undergo 10 days of intense technical, security, and cross-cultural training at CDC in Atlanta before being deployed on field assignments of 3–5 months duration. Additional training beyond the 10 days is provided for communication and data management volunteers; volunteers assigned to Nigeria, Pakistan, and Democratic Republic of Congo receive special management training.

## Scope of Volunteer Assignments

From January 1999 through June 2013, a total of 1,563 volunteers were identified, trained, and deployed for 2,221 STOP assignments to 69 countries.* Among those volunteers, 456 (23%) were from the United States (256 were CDC employees). The assignments included 1,802 field assignments, 217 communications assignments, and 202 data management assignments; 558 (25%) assignments were to polio endemic countries (Afghanistan, Nigeria, Pakistan, and previously, India). Among the assignments, 1,592 (72%) have been to English-speaking countries, 520 (23%) to French-speaking countries, and 109 (5%) to Portuguese-speaking countries. With the declaration of polio eradication as a programmatic emergency by the World Health Assembly in 2012, the number of STOP volunteers deployed increased from 90–120 per year during 1999–2011 to 287 in 2012 and 371 in 2013 (Figure); the number of volunteer person-months in the field per year increased from 273 in 1999 to 1,456 in 2012. Part of this growth in the number of person-months has resulted from an increasing number of volunteers serving on multiple assignments: 56% of the volunteers in 2013 had served in previous assignments. The STOP team deployed in February 2013 had 168 volunteers; the team deploying in July 2013 will be the largest to date, with an estimated 203 volunteers.

## Field Assignment Activities

In February 2013, a survey of 458 volunteers from STOP teams deployed at any time since February 2011 was conducted to assess activities conducted in field assignments. Among 312 (68%) volunteers who returned questionnaires, an average of 51% of their time deployed was reported to be spent on capacity-filling polio eradication activities, (e.g., active surveillance for AFP cases, AFP case verification, and updating supplemental immunization activity microplans), and an average of 49% of their time was spent on capacity-building activities (e.g., training health-care workers). The distribution of time spent on specific activities varied by country. For example, among 82 volunteers with field assignments in polio-endemic countries, an average of 68% of their time was reported to be spent conducting polio-related activities, 22% on routine childhood immunization strengthening activities, and 10% on other health programs. By comparison, among 230 volunteers with field assignments in nonendemic countries, an average of 54% of their time was spent on polio-related activities, 31% on routine immunization strengthening activities, and 15% on other health initiative activities.

What is already known on this topic?

Since the Global Polio Eradication Initiative (GPEI) was established in 1988, the annual incidence of polio has decreased >99%, and the number of countries in which wild poliovirus (WPV) circulation had never been interrupted has been reduced to three: Afghanistan, Nigeria, and Pakistan. The Stop Transmission of Polio (STOP) program was developed and initiated by CDC with the World Health Organization in 1999 to mobilize skilled personnel and technical resources to assist countries affected by WPV transmission.

What is added by this report?

The STOP program has become a large human resources deployment mechanism that has worked successfully for GPEI over an extended period. During 1999–2013, 1,563 volunteers were identified, trained, and deployed for 2,221 assignments as part of 42 STOP teams in 69 countries. The number and length of assignments has increased since the World Health Assembly declared the completion of polio eradication a programmatic emergency in 2012. The number of volunteers increased from 90–120 per year during 1999–2011 to 378 in 2013.

What are the implications for public health practice?

GPEI partnership will continue the STOP program throughout the period of eradication, certification, and progressive withdrawal of oral poliovirus vaccines, as outlined in the *Polio Eradication and Endgame Strategic Plan 2013–2018*. The STOP model has been replicated within Nigeria and Pakistan as a public health capacity-building mechanism; national field epidemiology programs have been recruiting, training, and deploying qualified national staff members to supervise and implement GPEI activities at country level. These staff members enhance national polio eradication programs overall, and particularly in areas that are not accessible to staff of international organizations because of security concerns.

### Editorial Note

The STOP program has made an important contribution to the mission of GPEI by providing countries with critical technical support to strengthen polio eradication activities. In response to requests from countries and WHO regional offices, STOP was expanded to provide a broader range of technical support for immunization programs, and the number of volunteers was increased over time. The flexibility of the STOP program enables volunteers to fill human resource gaps or build local capacity as needed by the country in which they are working. The effectiveness of STOP field assignments might be further enhanced through management training of some STOP supervisors and consistent development of clear work plans by field supervisors, in collaboration with STOP volunteers.

The STOP program concept has served as a model for training programs elsewhere. In Pakistan (since 2011) and in Nigeria (since 2012), for example, national STOP teams of local health professionals have been specifically trained to enhance the implementation of polio eradication activities. National STOP staff members aid national GPEI programs overall, and particularly in areas that are not accessible to staff of international organizations because of security concerns.

The STOP program is a coordinated effort of multiple GPEI partners. During 2000–2012, the Canadian Public Health Association, funded by the government of Canada, collaborated with CDC to identify, recruit, and deploy French-speaking participants for the STOP program. Rotary International and the Bill and Melinda Gates Foundation have contributed to funding for STOP volunteer field assignments. WHO and UNICEF organize field assignments through their regional and country offices. In addition, partners assist during the Atlanta-based training, providing technical and logistical support. The GPEI partnership will continue the STOP program throughout the period of eradication, certification, and progressive withdrawal of oral poliovirus vaccines, as outlined in the *Polio Eradication and Endgame Strategic Plan, 2013–2018* (7).

## Figures and Tables

**FIGURE:**
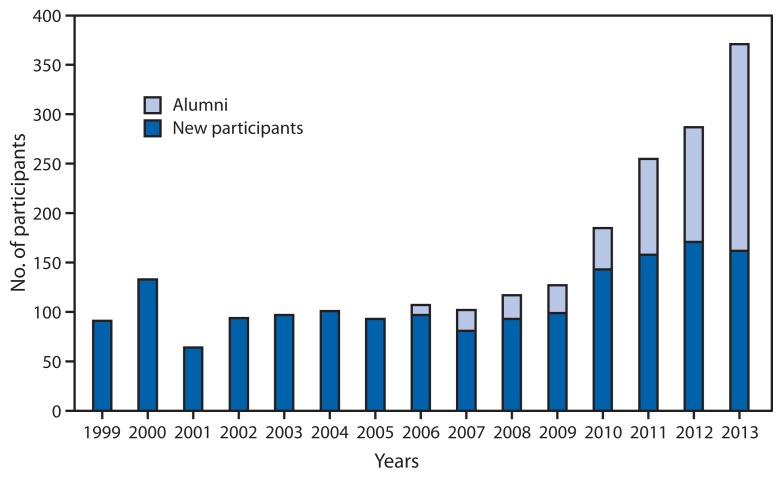
Annual numbers of new and returning Stop Transmission of Polio (STOP) volunteers — Global Polio Eradication Initiative, 1999–2013

## Footnotes

* Additional information on the STOP program and countries to which volunteers have been assigned (including map) is available at http://www.cdc.gov/polio/stop.
